# Construction and applications of the EOMA spheroid model of Kaposiform hemangioendothelioma

**DOI:** 10.1186/s13036-024-00417-4

**Published:** 2024-03-14

**Authors:** Yanan Li, Xinglong Zhu, Li Li, Chunjuan Bao, Qin Liu, Ning zhang, Ziyan He, Yi Ji, Ji Bao

**Affiliations:** 1 Department of Pediatric Surgery, Division of Oncology, West China Hospital of Sichuan University, 37# Guo-Xue-Xiang, Chengdu, 610041 China; 2 Med-X Center for Informatics, Sichuan University, Chengdu, 610041 China; 3 Department of Pathology, Institute of Clinical Pathology, Key Laboratory of Transplant Engineering and Immunology, West China Hospital, Sichuan University, 37# Guoxue Road, Chengdu, 610041 Sichuan Province China

**Keywords:** Kaposiform hemangioendothelioma, 3D cell model, EOMA spheroid, VEGFC, Sirolimus

## Abstract

**Background:**

Kaposiform hemangioendothelioma (KHE) is a rare intermediate vascular tumor with unclear pathogenesis. Recently, three dimensional (3D) cell spheroids and organoids have played an indispensable role in the study of many diseases, such as infantile hemangioma and non-involuting congenital hemangiomas. However, few research on KHE are based on the 3D model. This study aims to evaluate the 3D superiority, the similarity with KHE and the ability of drug evaluation of EOMA spheroids as an in vitro 3D KHE model.

**Results:**

After two days, relatively uniform morphology and high viability of EOMA spheroids were generated by the rotating cell culture system (RCCS). Through transcriptome analysis, compared with 2D EOMA cells, focal adhesion-related genes such as *Itgb4*, *Flt1*, *VEGFC*, *TNXB*, *LAMA3*, *VWF*, and *VEGFD* were upregulated in EOMA spheroids. Meanwhile, the EOMA spheroids injected into the subcutaneous showed more obvious KMP than 2D EOMA cells. Furthermore, EOMA spheroids possessed the similar characteristics to the KHE tissues and subcutaneous tumors, such as diagnostic markers (CD31 and LYVE-1), cell proliferation (Ki67), hypoxia (HIF-1α) and cell adhesion (E-cadherin and N-cadherin). Based on the EOMA spheroid model, we discovered that sirolimus, the first-line drug for treating KHE, could inhibit EOMA cell proliferation and downregulate the VEGFC expression. Through the extra addition of VEGFC, the effect of sirolimus on EOMA spheroid could be weakened.

**Conclusion:**

With a high degree of similarity of the KHE, 3D EOMA spheroids generated by the RCCS can be used as a in vitro model for basic researches of KHE, generating subcutaneous tumors and drug screening.

**Supplementary Information:**

The online version contains supplementary material available at 10.1186/s13036-024-00417-4.

## Introduction

Kaposiform hemangioendothelioma (KHE) is a rare vascular tumor, with a reported incidence rate of 0.9 cases per 100,000 children in the United States between 1991 and 2009 [1]. Initially described in 1993 by Zukerberg et al., KHE is characterized by locally aggressive behavior, consumptive coagulopathy leading to severe hypofibrinogenemia, a condition known as the Kasabach-Merritt phenomenon (KMP), and lymphangiomatosis [2, 3]. While KHE is typically categorized as a benign lesion, KHE with KMP poses a significant life-threatening risk to individuals affected by this condition. Reported mortality rates from 1991 to 2009 ranged from 12 to 30%, largely due to factors such as compression of vital structures, hemodynamic instability, and local invasion [1, 4, 5]. The pathophysiology of KHE is not yet completely known. Despite sharing a similar histology with Kaposi's sarcoma, KHE tissue does not positive for human herpesvirus 8, and its pathophysiology and behavior are distinct from that of Kaposi's sarcoma [6].The structural attributes of KHE are distinct and serve to increase turbulent blood flow and stimulate platelet activation, thereby contributing to its association with KMP [6]. Although no recurrent cytogenetic anomalies have been observed in KHE, Zhou et al. recently reported a balanced translocation t (13;16) (q14; p13.3) in a 7-year-old male patient with recurrent KHE [7].

Currently, many studies rely on traditional Two-dimensional (2D) in vitro cell culture or animal models for drug testing and exploring pathogenesis [8]. Both of these models, however, have insurmountable flaws. The 2D cell culture system differs significantly from in vivo tissue structure. The 2D cell culture system lacks the complex microenvironment of human tumors, including cell–cell, cell-extracellular matrix interactions, hypoxia, central necrosis and drug resistance, which are critical factors influencing cell fate and can lead to drug failure in clinical trials [9]. Three-dimensional (3D) tumor cell culture can considerably improve in vitro tumor cell vitality, histology, genotype stability, function, and drug metabolism [10]. A microtumor model of infantile hemangioma was effectively built, providing a more stable and efficient experimental model for investigating the mechanism and drug screening of infantile hemangioma [11]. Furthermore, animal models are unavoidably fraught with challenges such as high costs and ethical concerns.

Vascular endothelial growth factor C (VEGFC) contributes to tumor progression by affecting tumor cells directly or by regulating lymphangiogenesis and immune responses [12]. KHE is a consequence of a combination of angiogenesis and lymphangiogenesis imbalances, and VEGFC in lymphatic endothelial cells is essential throughout the lymphangiogenesis process [13]. Aside from lymphangiogenesis, the VEGFC/VEGFR-3 signaling pathway has been shown to be important for angiogenesis, working in tandem with the VEGFA/VEGFR-2 and Dll4/Notch signals to control angiogenesis [14, 15]. Efforts to produce anti-lymphangiogenic therapy for cancer based on VEGFC/D signaling offer great potential, and research into other targets is now underway [16]. In small animal models, such as zebrafish, the specific anti-lymphangiogenic effects of mTOR inhibition with rapamycin were observed, potentially mediated by the antagonism of VEGFC signaling [16]. VEGFC expression is significantly increased in KHE tissues, suggesting VEGFC may contribute to the development of KHE [17]. VEGFC may also stimulate the proliferation and invasion of tumor-associated immune cells like monocytes and macrophages, promoting the growth of KHE [18]. Furthermore, Flores et al. showed that sirolimus may exert anti-lymphangiogenic effects by inhibiting VEGFC [16]. The effectiveness of sirolimus treatment for KHE has been widely confirmed [19–21]. However, the precise mechanism of sirolimus in the treatment of KHE is still unknown.

In this study, with the benefits of 3D cell culture, we exploited the EOMA spheroids, as the model of KHE in vitro, generated by the dynamic 3D rotating cell culture system (RCCS). The generating process and functions of EOMA spheroids were observed and analysed. Changes in transcriptome were compared between 2D EOMA cells and 3D EOMA spheroids. Furthermore, we evaluated the ability of 2D EOMA cells and 3D EOMA spheroids to generate tumors in mice. The similarity of 3D EOMA spheroids, subcutaneous tumors and the KHE tissue was contrasted by the immunohistochemistry. Based on 3D EOMA spheroids (the in vitro model of KHE), the relationship between the effect of sirolimus and VEGFC was explored and The possible mechanism of sirolimus in treating KHE has also been analyzed.

## Materials and methods

### Cell culture

EOMA cells were cultured in the high-glucose Dulbecco's modified Eagle's medium (DMEM, HyClone, MA, USA), supplemented 1% penicillin–streptomycin solution (HyClone) and 10% foetal bovine serum (Gibco, NY, USA) in a 5% CO_2_ incubator (Thermo) at 37 °C.

### EOMA spheroids culture

Glass culture dishes (10 cm × 7.35 cm × 2 cm) were sterilized by autoclave sterilization after being treated with Sigmacote (Sigma, MO, USA) which was used for preventing cells adhering at dishes. EOMA cells were suspended in medium, then inoculated into a dish at cell density of 3 × 10^5^ cells/mL for 10 mL. The dishes were incubated at 37 °C in a humidified atmosphere and shaken at a rate of 10 times per minute for 3 days. On day 1, 2 and 3, the morphology of EOMA spheroids was observed and assessed by EVOS TM XL Core (Invitrogen). The diameter of EOMA spheroids was measured and analyzed by the ImageJ. EOMA spheroids were collected by the centrifugation for 5 min at the speed of 500 rpm. The cell number of EOMA spheroids was calculated by the measurement of DNA content. Briefly, collected EOMA spheroids and 1 × 10^6^ EOMA cells were used to extract DNA using the DNA extraction kit (Tiangen Biotech Corporation, Beijing, China). The samples were then quantified using a NanoDrop spectrophotometer (ND-2000c, Thermo). According to the total DNA content of 1 × 10^6^ EOMA cells, the cell number of EOMA spheroids was calculated. Their viability was examined by the Calcein-acetoxymethyl ester (Calcein AM)/propidium iodide (PI) cell live/dead assay kit (Solarbio, CA1630), the fluorescence was observed via the confocal microscopy. EOMA spheroids were fixed with 4% formaldehyde for 20 min at room temperature, stained with rhodamine phalloidin solution (100 nM) (Cytoskeleton) for 45 min at room temperature and counter-stained with DAPI working solution for 8 min, then were observed by the confocal microscopy.

### EOMA spheroids xenotransplantation

To compare the ability of forming tumors of 2D EOMA cells and 3D EOMA spheroids, 2D EOMA cells and 3D EOMA spheroids (1 × 10^6^ cells) resuspended in 100 μL Matrigel were respectively injected into the right flank of Balb/c nude mice (age: 6–8 weeks). Mice were sacrificed to collect the subcutaneous tumor and blood after 1 week. The platelet was analysed and counted.

### Histology and immunohistochemistry

The tumor markers, proliferative ability, hypoxic microenvironment and cell adhesion and interactions of EOMA spheroids and EOMA subcutaneous tumor tissue and KHE tissue were measured and compared through immunohistochemistry. EOMA spheroids, EOMA subcutaneous tumor tissue and KHE tissue were fixed in 4% neutral formalin at room temperature for 24 h, then were dehydrated by a graded ethanol series, immersed in xylene and embedded in paraffin. Samples were further cut into 4 μm sections and stained with hematoxylin and eosin (H&E) and primary antibodies against Ki-67 (ab16667, 1:100, Abcam), HIF-1α (ab51608, 1:100, Abcam), N-cadherin (66,219–1-Ig, 1:100, Proteintech) and E-cadherin (ab231303, 1:200, Abcam).

### RNA sequencing

The total RNA was respectively extracted from 2D EOMA cells and 3D EOMA spheroids and their integrity and quality were assessed. The library products, ranging from 200 to 500 bps, were enriched, quantified, and sequenced using Illumina's NovaSeq 6000 outfitted with the PE150 model after RNA library preparation and DNA cluster production were finished.

### Quantitative real-time polymerase chain reaction (qRT–PCR)

The total RNA was respectively extracted from after 2 days culture of 2D EOMA cells and 3D EOMA spheroids by TRIzol (15,596–026, Invitrogen). Complementary DNA (cDNA) was synthesised using the iScript cDNA Synthesis Kit (Bio-Rad). The glyceraldehyde-3-phosphate dehydrogenase (*GAPDH*) was served as an endogenous internal control. The PCR reactions were performed in triplicate followed by gene expression analysis and quantification using the Stratagene analysis software and the 2^−ΔΔCt^ method, respectively (primer sequences were provided in Table S1).

### RNA-seq data analysis

The raw sequencing data underwent initial filtration using Trimmomatic (version 0.36) to discard low-quality reads and trim sequences contaminated with adaptors. Subsequently, clean reads underwent additional processing with custom scripts to mitigate duplication bias stemming from library preparation and sequencing. Initially, clean reads were grouped based on unique molecular identifier (UMI) sequences, clustering reads with identical UMIs together. Within each cluster, pairwise alignment was conducted to identify reads with sequence identity exceeding 95%, which were then organized into new sub-clusters. Following the generation of all sub-clusters, multiple sequence alignment was performed to derive a consensus sequence for each sub-cluster. These procedures effectively minimized errors and biases arising from PCR amplification or sequencing.

The consensus sequences, after deduplication, were employed for standard RNA-seq analysis. The sequences were aligned to the mouse reference genome employing STAR software (version 2.5.3a) using default settings. Subsequently, reads mapped to gene exon regions underwent counting via featureCounts (Subread-1.5.1; Bioconductor), followed by RPKM computation. Differential gene expression between experimental groups was determined using the edgeR package (version 3.12.1), applying a significance threshold of p-value ≤ 0.05 and fold-change cutoff of 2. Enrichment analyses for differentially expressed genes, involving Gene Ontology (GO) and Kyoto Encyclopedia of Genes and Genomes (KEGG), were performed using KOBAS software (version: 2.1.1), with a significance cutoff of p-value ≤ 0.05. Detection of alternative splicing events was executed utilizing rMATS (version 3.2.5), with an FDR value cutoff of 0.05 and an absolute value of Δψ of 0.05.

### Evaluation of drug effects

After 2 days shacking culture, EOMA spheroids were collected, then seeded into the 96-well culture plate at a density of 1 × 10^4^ cells per well. The cell number of EOMA spheroids was calculated by the measurement of DNA content. Furthermore, EOMA spheroids were treated with sirolimus (5nM, 20nM, 50nM, and 100nM) and VEGFC (5ng/mL, 20ng/mL, 50ng/mL, and 100ng/mL) for 48 h, respectively. For further studying the effect of VEGFC to intervention of EOMA spheroids with sirolimus. EOMA spheroids were simultaneously treated with the combination of sirolimus (50nM) and different concentrations of VEGFC (0ng/mL, 5ng/mL, 20ng/mL, 50ng/mL) for 48 h. The cell viability was assessed by the CCK-8. For explore the sprouting ability of EOMA spheroids, EOMA spheroids (5 × 10^4^ cells) were transferred into 50 μL Matrigel, which then treated with sirolimus (50nM), VEGFC (20ng/mL) and sirolimus (50nM) + VEGFC (20ng/mL), respectively. The EOMA spheroid sprouting was observed and analysed after 24 and 48 h of treatment.

### Western blot analysis

Briefly, the protein concentration was determined using the Bradford protein assay kit (Bio-Rad). Then the protein samples were separated by sodium dodecy1 sulphate–polyacrylamide gel electrophoresis, followed by electrophoretically transferred onto a nitrocellulose membrane. The membrane was incubated with primary antibody in TBST at 4℃ overnight. Next, the membrane was washed three times and incubated with the appropriate secondary antibody. The protein bands were visualized using enhanced ECL-associated fluorography. The following antibodies were from Abcam: VEGFC (1:1000 dilution for WB) and ACTB/β-actin (1:100,000 dilution for WB).

### Statistical analysis

For statistical analysis, this study used SPSS 21.0 software (SPSS, Inc., Chicago, USA). The mean and standard deviation for all quantitative values was displayed. For a quantitative analysis, the Dunnett's test was applied. For multiple statistical tests, ANOVA was used. Statistical significance was defined as any p-value less than 0.05. The analysis of the RNA-Seq data was performed using the R software version 4.1.0.

## Result

### Rapid generation of 3D EOMA spheroid model using RCCS

**Fig. 1:**
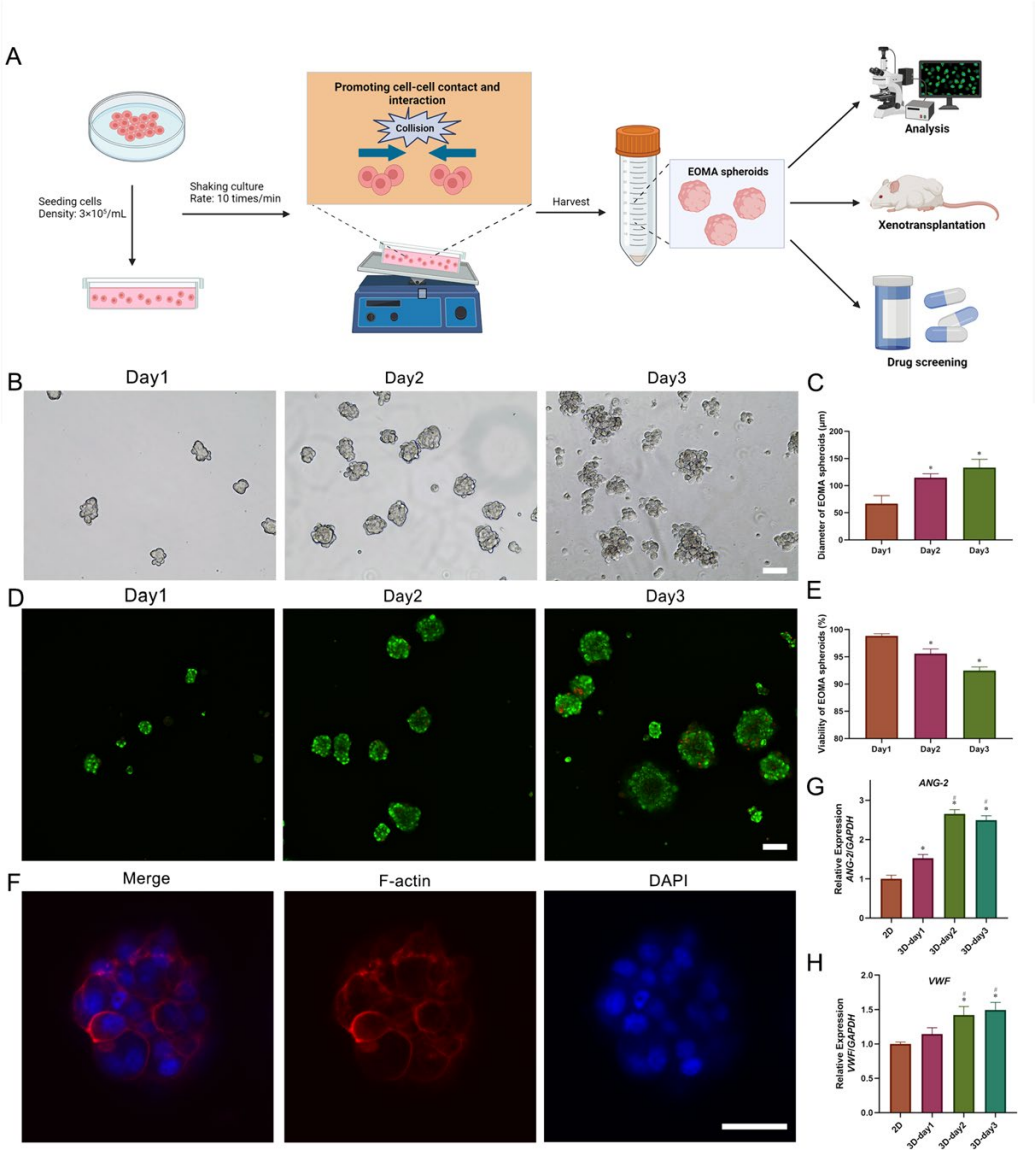
Fabrication of EOMA spheroids. **A** Schematic showing formation process and mechanism and applications of EOMA spheroids. **B** The morphology of EOMA spheroids at day 1, 2 and 3. **C** The diameter of EOMA spheroids at day 1, 2 and 3. **D** Live/Dead staining of EOMA spheroids at day 1, 2 and 3. **E** The viability of EOMA spheroids at day1, 2 and 3. **F** Phalloidine staining of EOMA spheroids at day 2. **G** The expression of *ANG-2* in 2D EOMA cells at day 2 and EOMA spheroids at day 1, 2 and 3. **H** The expression of *VWF* in 2D EOMA cells at day 2 and EOMA spheroids at day 1, 2 and 3. *P < 0.05, compared to the 2D culture. *#P* < 0.05, compared to 3D-day1. Scale bars = 50 μm

The RCCS contained a shaker with multilayer and glass culture dishes (Figure S1). We observed the formation of spherical multicellular aggregates with an average diameter of approximately 66.87 ± 13.2 μm after shaking EOMA cells in RCCS for one day. After two days, reasonably uniform 3D cell spheroids with an average diameter of roughly 105.4 ± 15.1 μm formed. After three days, the cell spheroids continued to grow in size, with some fusing together, and an average diameter of around 110.5 ± 24.8 μm formed (Fig. 1B and C). The FluoroQuench results revealed that the cell viability was 92.2 ± 0.5%, 91.6 ± 0.9% and 87.6 ± 0.6% at day 1, day 2 and day 3, respectively (Fig. 1D and E). The expression of two endothelial-related genes (*ANG-2* and *VWF*) was detected by qRT-PCR. *ANG-2* and *VWF* were expressed at significantly higher levels in 3D spheroids than in after 2 days of culture 2D cells. In addition, the expression levels of *VWF* and *ANG-2* were significantly higher in EOMA spheroids at day 2 and day 3 than in EOMA cell spheroids at day 1 (Fig. 1E and G). After shaking and cultured for two days, EOMA spheroids shown the spherical characteristic (Fig. 1F).

### Contrasting the functions of 3D EOMA spheroid and 2D EOMA cells by transcriptome sequencing

**Fig. 2:**
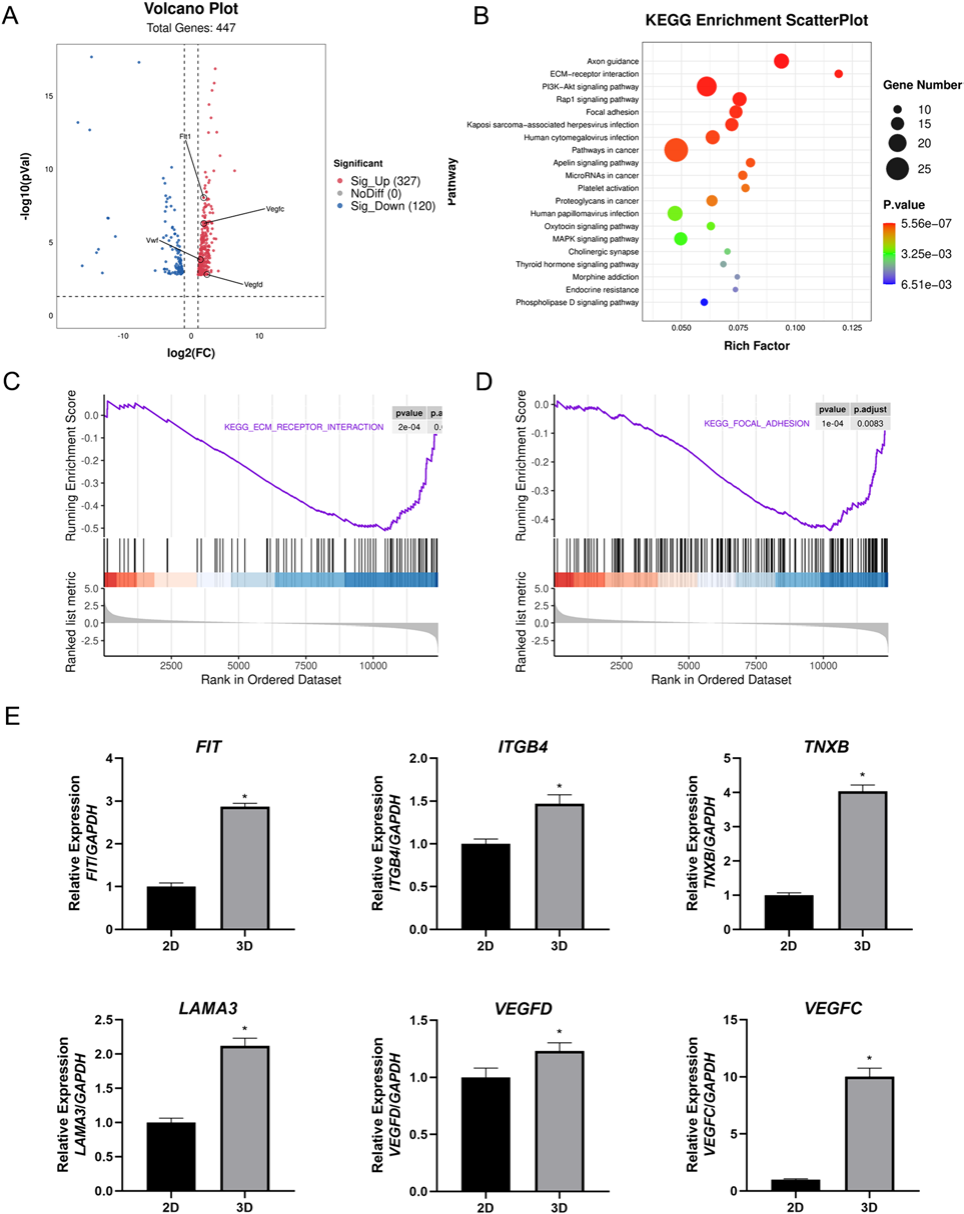
The results of RNA sequencing. **A** Volcano plot, High relative expression is indicated by red, whereas low relative expression is indicated by blue. **B** The KEGG signaling pathways. **C** The GSEA analysis of ECM -receptor interaction pathway. **D** The GSEA analysis of Focal adhesion signaling pathway. **E** Gene expression levels of focal adhesion-related genes (*ITGB4*, *Flt1*, *VEGFC*, *TNXB*, *LAMA3* and *VEGFD*). *P < 0.05, compared to the 2D culture

Transcriptome sequencing was conducted on EOMA spheroid and 2D cells to investigate gene changes during EOMA spheroid formation, showing 327 significantly upregulated genes and 120 significantly downregulated genes (Fig. 2A). Angiogenesis, branching engaged in blood vessel morphogenesis, positive regulation of angiogenesis, positive regulation of cell migration, and sprouting angiogenesis were all found to be significantly enriched in GO enrichment analysis (Figure S2. The ECM-receptor interaction pathway, the PI3K-Akt signaling pathway, focal adhesion, and platelet activation were all significantly enriched in the KEGG pathway enrichment study (Fig. 2B). The focal adhesion signaling pathway serves as a crucial mechanism governing cell adhesion, migration, proliferation, and differentiation through intricate interactions with the extracellular matrix (ECM) [22]. This signaling cascade plays a pivotal role in orchestrating cellular responses to the external environment, intricately influencing various aspects of cell behavior and function. The focal adhesion signaling pathway emerges as a pivotal player in angiogenesis, exerting its influence on the adhesion and migration dynamics of vascular endothelial cells [23]. The GSEA analysis of ECM -receptor interaction and Focal adhesion signaling pathway were shown in Fig. 2C and Fig. 2D. qRT-PCR confirmed that focal adhesion-related genes such as *Itgb4*, *Flt1*, *VEGFC*, *TNXB*, *LAMA3*, *VWF*, and *VEGFD* were substantially upregulated (Figs. 1H and 2E).

### The ability of EOMA spheroids contributing to generate tumor in immunodeficient mice

**Fig. 3:**
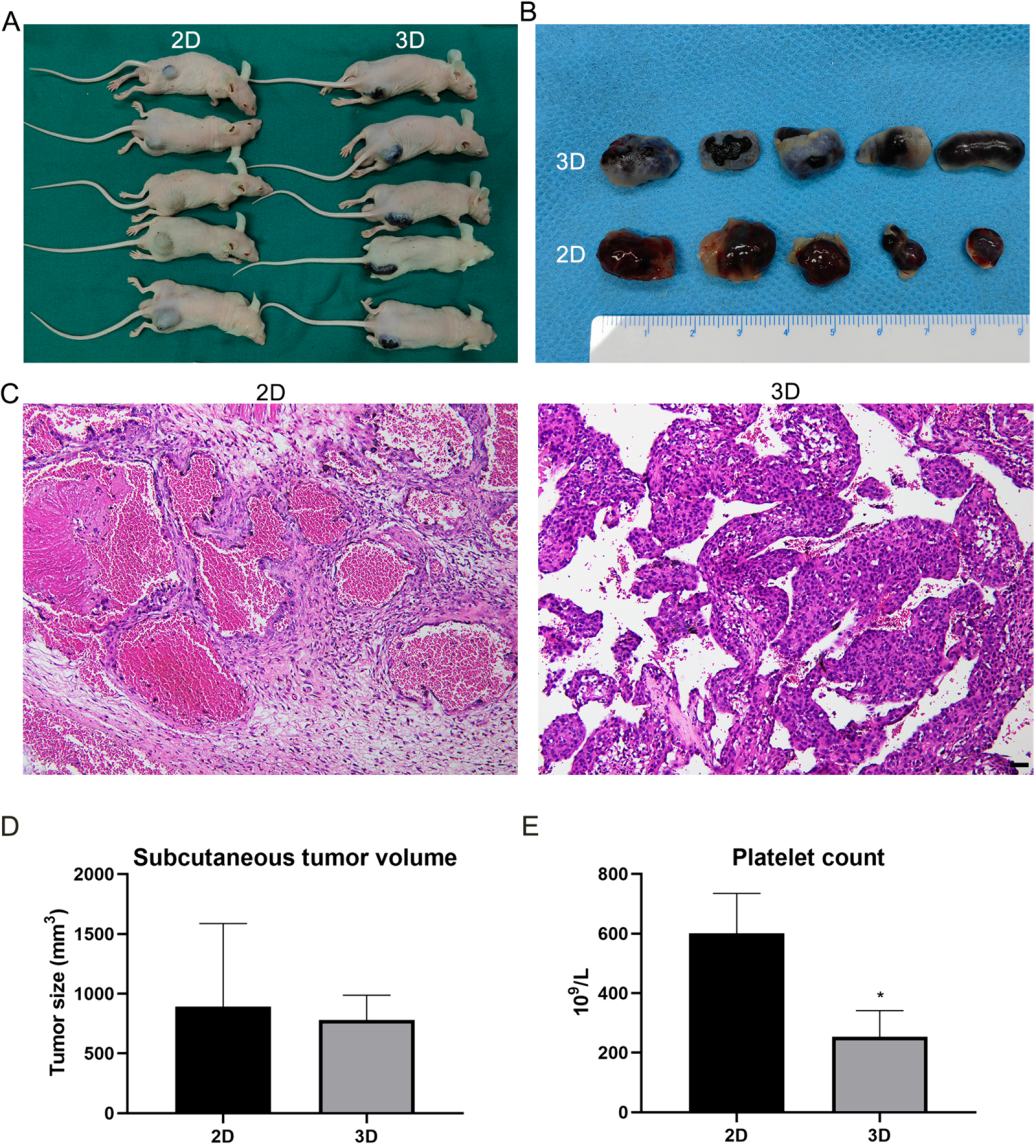
EOMA spheroids xenotransplantation. **A** The image of subcutaneous tumor model in nude mice with injected with 3D EOMA spheroids than 2D EOMA cells. **B** The image of subcutaneous tumors. **C** The H&E of subcutaneous tumors. **D** The tumor volume of subcutaneous tumors. **E** The platelet count. *P < 0.05, compared to 2D

To evaluate the ability of EOMA spheroids to generate tumors in mice, the 2D EOMA cells and 3D EOMA spheroids were collected, then mixed with matrixgel, and inoculated subcutaneously into the right abdomen of nude mice. After one week, the 2D EOMA cells and 3D EOMA spheroids possessed the ability to form the tumors in mice (Fig. 3A and B). Interestingly, the KMP was more easily to occur in mice with injected with 3D EOMA spheroids than 2D EOMA cells (Fig. 3A). Meanwhile, the routine analysis of blood manifested that the platelets were more significantly reduced in the mice injected with 3D EOMA spheroids than 2D EOMA cells (Fig. 3E). the volume of tumors was further contrasted, the experimental result shown that the average volume of tumors were bigger in mice with injected with 3D EOMA spheroids than 2D EOMA cells but there is no significance (Fig. 3D). It's worth noting that the uniformity of tumors was better in in mice with injected with 3D EOMA spheroids than 2D EOMA cells (Fig. 3B and D). HE staining confirmed that tumor cells were grew actively and densely arrayed in mice with injected with 3D EOMA spheroids than 2D EOMA cells after subcutaneous tumor formation (Fig. 3C).

### Characteristics of the cell spheroid model

**Fig. 4:**
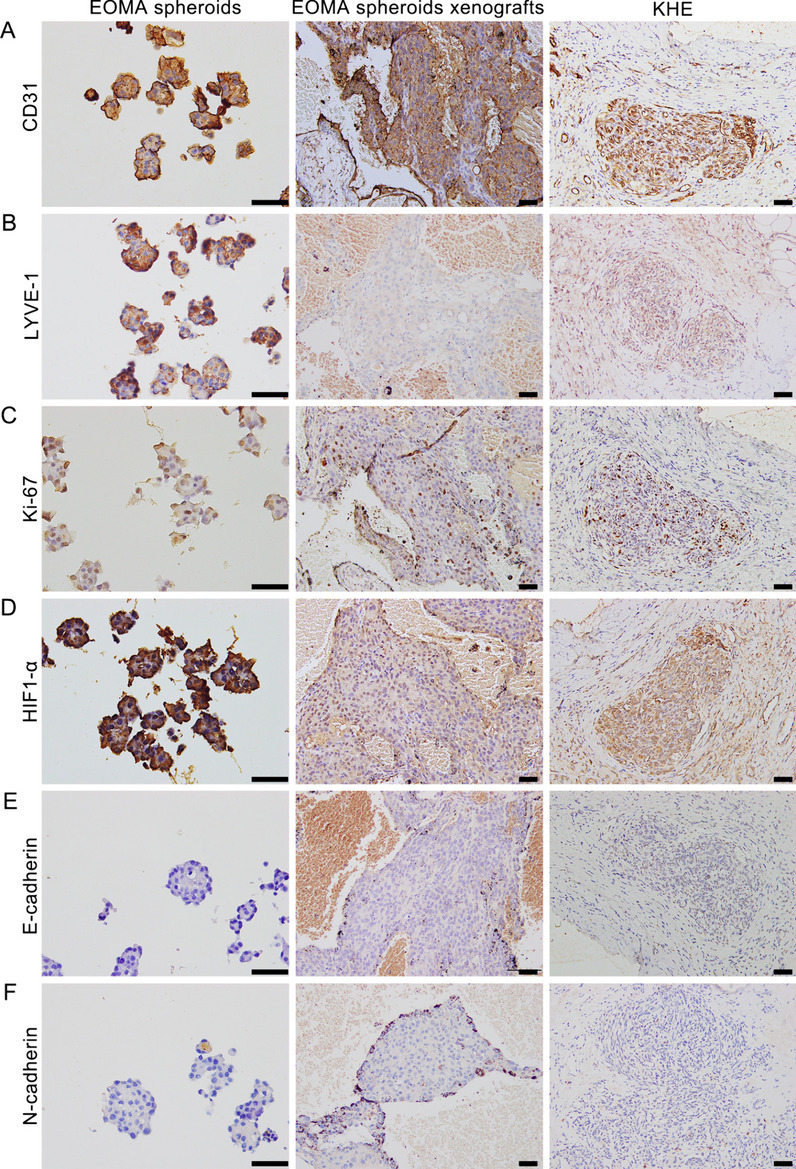
The similarity of EOMA spheroids to subcutaneous tumors and KHE tissue. **A** Immunohistochemistry staining of CD31. **B** Immunohistochemistry staining of LYVE-1. **C** Immunohistochemistry staining of Ki67. **D** Immunohistochemistry staining of HIF-1α, (**E**) Immunohistochemistry staining of E-cadherin, (**F**) Immunohistochemistry staining of N-cadherin. Scale bars = 100 μm

EOMA spheroids after shaking and cultured for two days and tumors in mice with injected with 3D EOMA spheroids were respectively collected for further study. The similarity of EOMA spheroids to subcutaneous tumors and KHE tissue was assessed by immunohistochemistry. The tumor markers of KHE, such as platelet endothelial cell adhesion molecule-1 (CD31) and LYVE-1, were evaluated. The CD31 was expressed similarly in all three groups (Fig. 4A). However, the LYVE-1 was found to be strongly expressed in EOMA spheroids and KHE tissues, but not in subcutaneous tumors (Fig. 2B). The proliferative marker Ki67 and the hypoxia marker hypoxia inducible factor 1α(HIF-1α) were all expressed similarly in all three groups (Fig. 2C and D). Furthermore, neither E-cadherin nor N-cadherin were substantially expressed in any of the three groups (Fig. 2E and F). In general, EOMA spheroids possessed the similar characteristics to the KHE tissues and subcutaneous tumors.

### The effect of sirolimus on EOMA spheroids

**Fig. 5:**
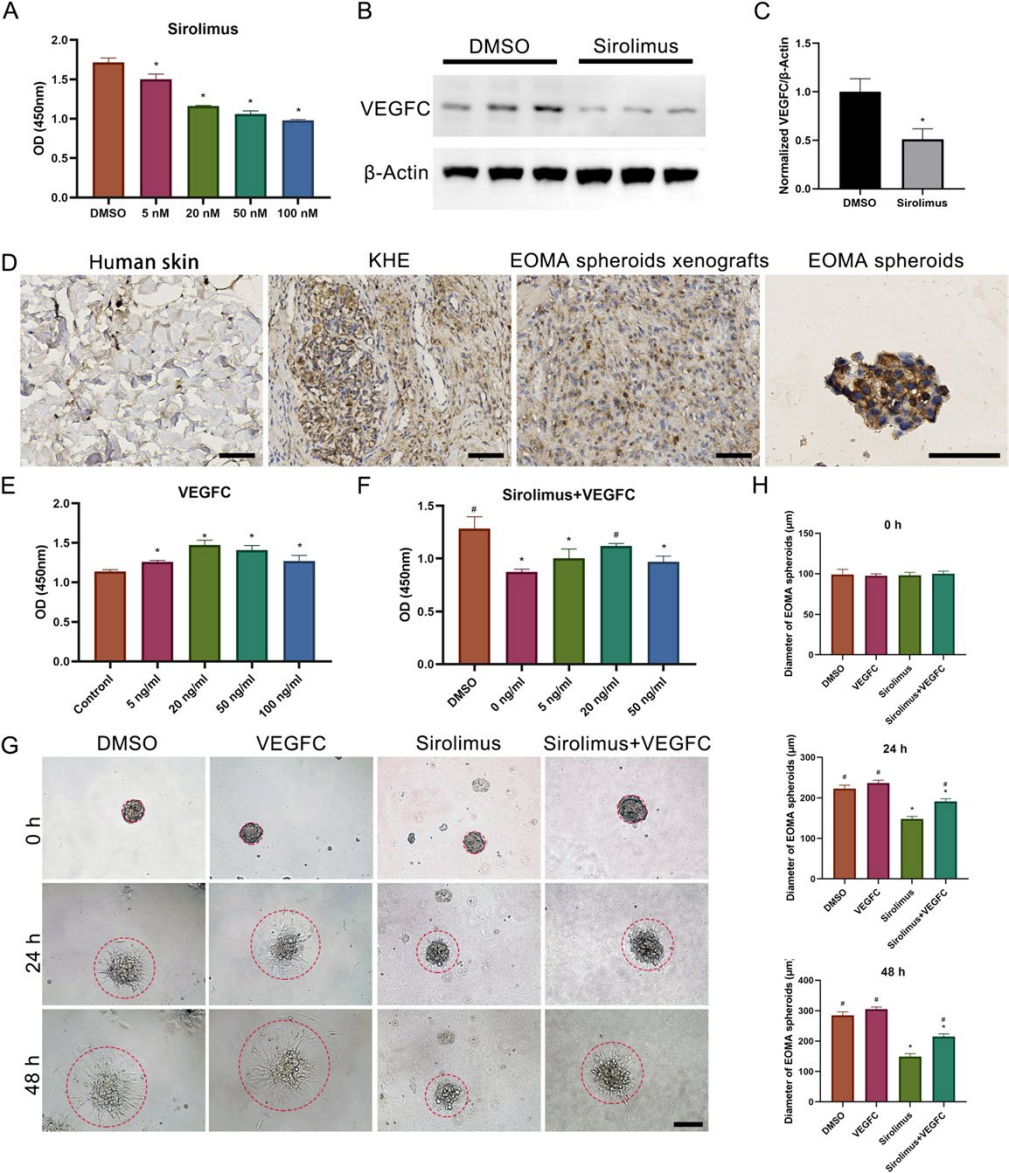
The effect of sirolimus on EOMA spheroids. **A** The proliferation of EOMA spheroids treated with sirolimus for 24 h. **B** Western blot of expression levels of VEGFC at EOMA spheroids treated with DMSO and sirolimus. **C** The WB semiquantitative analysis. **D** Immunohistochemistry staining of VEGFC in normal human skin tissue, KHE tissue, subcutaneous tumors and EOMA spheroids. **E** The proliferation of EOMA spheroids treated with VEGFC for 24 h. **F** The intervention of EOMA spheroids treated with sirolimus by VEGFC. **G** The sprouting of EOMA spheroids treated with sirolimus and VEGFC for 0, 24 and 48 h. **H** The diameter of EOMA spheroids treated with sirolimus and VEGFC for 0, 24 and 48 h. *P < 0.05, compared to the DMSO or control group (**A**, **C** and **E**). *P < 0.05, compared to the DMSO, *#P* < 0.05, compared to 50nM sirolimus (**F** and **H**). Scale bars = 100 μm

After shaking and cultured for two days, EOMA spheroids were collected, then treated with sirolimus (5nM, 20nM, 50nM, and 100nM). The CCK-8 result shown that compared with DMSO group, the sirolimus (5nM, 20nM, 50nM, and 100nM) could inhibit the EOMA cell proliferation, especially in the concentration of sirolimus over 20nM (Fig. 5A). Therefore, the 50nM sirolimus was selected for further study. EOMA spheroids treated with EOMA spheroids or DMSO were used to extract protein for WB. Flores et al. showed that sirolimus may exert anti-lymphangiogenic effects by inhibiting VEGFC [16]. We founded that the VEGFC expression was higher in DMSO group than 50nM sirolimus group (Fig. 5B and C). Furthermore, immunohistochemistry demonstrated that VEGFC was considerably more abundant in KHE than in adjacent tissues. VEGFC was highly expressed in EOMA spheroids, subcutaneous tumors and KHE than normal human skin tissue (Fig. 5D). These results indicated that the VEGFC could be likely very important to KHE development and sirolimus inhibited the EOMA cell proliferation probably via reducing the VEGFC expression. To test these ideas, EOMA spheroids were treated with VEGFC (5ng/mL, 20ng/mL, 50ng/mL, and 100ng/mL). The CCK-8 result shown that compared with the control, the VEGFC (5ng/mL, 20ng/mL, 50ng/mL, and 100ng/mL) could promote the EOMA cell proliferation, especially in the concentration of VEGFC over 20ng/mL (Fig. 5E). For studying the intervention of EOMA spheroids treated with 50nM sirolimus by adding different concentrations of VEGFC (5ng/mL, 20ng/mL, 50ng/mL), 20ng/mL VEGFC could significantly reverse the inhibitory effect of sirolimus on EOMA spheroids (Fig. 5F). The sprouting of EOMA spheroids could be greatly inhibited by 50nM sirolimus. Inhibitory effect of sirolimus on EOMA sprouting can be slightly reversed by VEGFC (20ng/mL) (Fig. 5G and H).

## Discussion

Obtaining a suitable model to summarize the complex tumor-host interactions is essential for comprehending tumor biology and devising effective therapeutic strategies [24]. 3D cell culture can provide cells with a physiological environment that is closer to nature, allowing cells to better simulate their functions and interactions in vivo and forecast their behavior in vivo with higher precision [25]. In order to improve the modeling of KHE, an ailment for which our understanding of the pathological and therapeutic mechanisms is presently restricted, we generated a 3D EOMA spheroid model utilizing the RCCS. We successfully constructed a 3D-EOMA cell spheroids model and found 327 significantly upregulated genes and 120 significantly downregulated genes during spheroid formation by transcriptome sequencing. Compared to 2D Culture, A number of pro-tumoral genes were substantially upregulated. By immunohistochemistry, we found that the 3D EOMA spheroid model and the EOMA subcutaneous xenograft tumor model of nude mice shared some similarity with the KHE tissue.

Several methods have been developed to generate 3D cell spheroids, such as scaffold-based methods (matrix encapsulation and spinner flasks) and scaffold-free methods (hanging drop and microwell method) [26]. Generally, all of these methods can be used to culture tumor spheroid with multicellular structure and cell–cell interactions and regulations of various substrates make tumor spheroids more similar to the drug response in vivo, but have their respective characteristics and advantages [26, 27]. Hydrogel is mainly used to supply the scaffold and ECM for supporting organization of tumor cells in the matrix encapsulation method. Although hydrogel can supply external cues which boost cell–cell and cell-ECM interaction but tumor spheroids with heterogeneous shape and size are difficult to observe and analyze in hydrogel. The operation and cost of matrix encapsulation are tedious and expensive and culture time is often very long, over 4–5 days [26]. The hanging drop takes advantage of the gravitational force to induce organization of tumor cells and the microwell method uses low attachment plates to reduce the cell − substrate interaction for generating tumor spheroids. These methods are low-cost and broad-spectrum and need short period of time (1–3 days). However, the operation is tedious, tumor spheroids is hard to collect and it is difficult to achieve on a large scale [25, 28]. Spinner flasks and RCCS are based on the same type of generating spheroids (shaking system). Through rotating and rocking, cell–cell contact and interaction are promoted to accelerate the self-aggregation of tumor cells. The shaking culture provides the dynamic microenvironment which improves the exchange efficiency of substances and is conducive to cell survival and large-scale culture. Moreover, the collection of tumor spheroids is simple and culture time is short period of time (1–3 days), which is favorable for the application of tumor spheroids. The RCCS has been used to culture large-scale hepatocyte spheroids (10^10^ cells) [29]. Consequently, each method has its advantages and disadvantages. The RCCS can efficiently generate tumor spheroids for drug screening and basic research.

Approximately 44% to 71% of cases of KHE are accompanied by life-threatening consumptive coagulopathy and severe thrombocytopenia [30]. Platelet trapping can be seen histologically in KHE with or without KMP [31]. Platelet activation can result in the release of growth factors such as transforming growth factor (TGF) and VEGF, both of which can increase the growth and proliferation of endothelial and tumor cells [32]. Platelet activation in KHE patients may result in platelet aggregation and thrombus development, resulting in vascular inflammation and tumor progression [18]. In our study, we found that after EOMA formed spheroids, the platelet activation signaling pathway was significantly enriched. The Focal adhesion signal pathway is a signaling mechanism that regulates cell adhesion, migration, proliferation, and differentiation by interacting with the extracellular matrix (ECM) [22]. The Focal adhesion signal pathway can play a role in angiogenesis by influencing the adhesion and migration of vascular endothelial cells [23]. The Focal adhesion signal pathway can regulate cell survival, proliferation and differentiation by activating various downstream signaling pathways, such as Rho GTPases, MAPK and PI3K/AKT [23]. In our research, we found that after EOMA spherogenesis, focal adhesion-related genes, including *ITGB4*, *FLT1*, *VEGFC*, *TNXB*, *LAMA3*, *VWF* and *VEGFD*, were significantly upregulated. The genes *FLT1*, *VEGFC*, *VWF*, and *VEGFD* are closely linked to angiogenesis and vascular function, playing important roles in tumor angiogenesis. Their positive expression in KHE indicates their potential contribution to the angiogenesis and tumor progression of this condition [33, 34]. Related signaling pathways, including ECM-receptor interaction pathway, PI3K-Akt signaling pathway, and MAPK signaling pathway, were highly enriched. The Focal adhesion signaling pathway may play an important role in the development of KHE.

The overexpression of VEGFR-3 in KHE may cause aberrant endotheslial cell proliferation, and thereby, accelerating the development of tumors [17, 35, 36]. VEGFR-3 is the main receptor for VEGFC and VEGFD and is involved in regulating endothelial cell proliferation, migration, and angiogenesis [37]. In our research, we discovered that in EOMA cell spheroids, both VEGFC and VEGFD were substantially upregulated. Additional immunohistochemical testing supported the finding that VEGFC was markedly elevated in KHE tissues. VEGFC has been shown to stimulate tumor cell growth and migration, as well as enhance neovascularization [38]. High VEGFC expression is associated with poor prognosis and metastasis in some kinds of tumors [39]. In our research, we discovered that VEGFC can substantially promote EOMA proliferation.

The Food and Drug Administration of the United States has not yet approved any pharmaceuticals to treat KHE [21]. Consensus treatment protocols recommend the administration of corticosteroids and/or vincristine [40]. However, many patients did not respond to corticosteroid monotherapy [41]. Furthermore, vincristine has been demonstrated to be ineffective in some severe KHE cases [42]. Since 2010, an increasing number of studies have documented sirolimus's unique therapeutic effects in the treatment of KHE [30, 43, 44]. In our previous studies, the effectiveness and safety of sirolimus in treating KHE were confirmed through short-term and long-term follow-up [19, 20]. We showed in a clinical randomized controlled trial that the combination of sirolimus and prednisolone is extremely effective in treating KHE combined with KMP [21]. Sirolimus is a drug that inhibits the mammalian target of rapamycin protein, which is important in cell development and survival. However, the mechanism of sirolimus in treating KHE is largely unknown. In the present study, we demonstrated that sirolimus can inhibit the proliferation of EOMA by downregulating the expression of VEGFC, and the addition of VEGFC can effectively rescue the inhibition of sirolimus on EOMA. Sirolimus may exert its effects in the treatment of KHE by inhibiting the expression of VEGFC.

## Conclusion

The RCCS could be used to rapidly generate 3D EOMA spheroids as the in vitro KHE model. Compared to the 2D EOMA cells, focal adhesion-related genes were upregulated in 3D EOMA spheroids. Furthermore, EOMA spheroids injected into the subcutaneous showed more obvious KMP than 2D EOMA cells. Characteristics of EOMA spheroids were highly similar to subcutaneous tumors and the KHE tissue. Based on the EOMA spheroid model, we discovered that sirolimus, the first-line drug for treating KHE, could inhibit EOMA cell proliferation and be downregulated the VEGFC expression. Through the extra addition of VEGFC, the effect of sirolimus on EOMA spheroid could be weakened. In conclusion, with a high degree of similarity of the KHE, 3D EOMA spheroids generated by the RCCS can be used as a in vitro model for basic researches of KHE, generating subcutaneous tumors and drug screening. VEGFC may play an important role in the development of KHE, and sirolimus may inhibit the progression of KHE by reducing the expression of VEGFC.

### Supplementary Information

**Supplementary Material 1.**

## Data Availability

No datasets were generated or analysed during the current study.

## Abbreviations

KHE: Kaposiform hemangioendothelioma
RCCS: Rotating cell culture system
KMP: Kasabach-Merritt phenomenon
2D: Two-dimensional
3D: Three-dimensional
VEGFC: Vascular endothelial growth factor C
EOMA: Hemangioendothelioma
H&E: Hematoxylin and eosin
GAPDH: Glyceraldehyde-3-phosphate dehydrogenase
HIF-1: Hypoxia inducible factor 1
TGF: Transforming growth factor
ECM: Extracellular matrix

## References

[1] Croteau SE, Liang MG, Kozakewich HP, Alomari AI, Fishman SJ, Mulliken JB, Trenor CC (2013). Kaposiform hemangioendothelioma: atypical features and risks of Kasabach-Merritt phenomenon in 107 referrals. J Pediatr.

[2] Li X, Wen MZ, Su LX, Yang XT, Han YF, Fan XD (2019). Local suture ligation-assisted percutaneous sclerotherapy for Kasabach-Merritt phenomenon-associated kaposiform haemangioendothelioma. Oncol Lett.

[3] Zukerberg LR, Nickoloff BJ, Weiss SW (1993). Kaposiform hemangioendothelioma of infancy and childhood An aggressive neoplasm associated with Kasabach-Merritt syndrome and lymphangiomatosis. Am J Surg Pathol.

[4] Yadav D, Maheshwari A, Aneja S, Seth A, Chandra J (2011). Neonatal Kasabach-Merritt phenomenon. Indian J Med Paediatr Oncol.

[5] Wang D, Chen X, Li Z (2019). Population pharmacokinetics of sirolimus in pediatric patients with kaposiform hemangioendothelioma: A retrospective study. Oncol Lett.

[6] Putra J, Gupta A (2017). Kaposiform haemangioendothelioma: a review with emphasis on histological differential diagnosis. Pathology.

[7] Zhou S, Wang L, Panossian A, Anselmo D, Wu S, Venkatramani R (2016). Refractory Kaposiform Hemangioendothelioma Associated with the Chromosomal Translocation t(13;16)(q14;p13.3). Pediatr Dev Pathol.

[8] Zhu X, Li Y, Yang Y, He Y, Gao M, Peng W, Wu Q, Zhang G, Zhou Y, Chen F (2022). Ordered micropattern arrays fabricated by lung-derived dECM hydrogels for chemotherapeutic drug screening. Mater Today Bio.

[9] Yang L, Yang S, Li X, Li B, Li Y, Zhang X, Ma Y, Peng X, Jin H, Fan Q (2019). Tumor organoids: From inception to future in cancer research. Cancer Lett.

[10] Xiang L, Yin Y, Zheng Y, Ma Y, Li Y, Zhao Z, Guo J, Ai Z, Niu Y, Duan K (2020). A developmental landscape of 3D-cultured human pre-gastrulation embryos. Nature.

[11] Li Y, Zhu X, Kong M, Chen S, Bao J, Ji Y. Three-dimensional microtumor formation of infantile hemangioma-derived endothelial cells for mechanistic exploration and drug screening. Pharmaceuticals (Basel). 2022;15(11):1393. 10.3390/ph15111393 PMC9692769 36422523

[12] Karpanen T, Egeblad M, Karkkainen MJ, Kubo H, Yla-Herttuala S, Jaattela M, Alitalo K (2001). Vascular endothelial growth factor C promotes tumor lymphangiogenesis and intralymphatic tumor growth. Cancer Res.

[13] Flister MJ, Wilber A, Hall KL, Iwata C, Miyazono K, Nisato RE, Pepper MS, Zawieja DC, Ran S (2010). Inflammation induces lymphangiogenesis through up-regulation of VEGFR-3 mediated by NF-kappaB and Prox1. Blood.

[14] Hsu MC, Pan MR, Hung WC. Two birds, one stone: double hits on tumor growth and lymphangiogenesis by targeting vascular endothelial growth factor receptor 3. Cells. 2019;8(3):270. 10.3390/cells8030270 PMC6468620 30901976

[15] Varricchi G, Granata F, Loffredo S, Genovese A, Marone G (2015). Angiogenesis and lymphangiogenesis in inflammatory skin disorders. J Am Acad Dermatol.

[16] Flores MV, Hall CJ, Crosier KE, Crosier PS (2010). Visualization of embryonic lymphangiogenesis advances the use of the zebrafish model for research in cancer and lymphatic pathologies. Dev Dyn.

[17] Saito M, Gunji Y, Kashii Y, Odaka J, Yamauchi T, Kanai N, Momoi MY (2009). Refractory kaposiform hemangioendothelioma that expressed vascular endothelial growth factor receptor (VEGFR)-2 and VEGFR-3: a case report. J Pediatr Hematol Oncol.

[18] Ji Y, Chen S, Yang K, Xia C, Li L (2020). Kaposiform hemangioendothelioma: current knowledge and future perspectives. Orphanet J Rare Dis.

[19] Zhou J, Li Y, Qiu T, Gong X, Yang K, Zhang X, Zhang Z, Lan Y, Hu F, Peng Q, Zhang Y, Kong F, Chen S, Ji Y. Long-term outcomes of sirolimus treatment for kaposiform hemangioendothelioma: Continuing successes and ongoing challenges. Int J Cancer. 2023;153(3):600–8. 10.1002/ijc.34509 36916140

[20] Ji Y, Chen S, Xiang B, Li K, Xu Z, Yao W, Lu G, Liu X, Xia C, Wang Q (2017). Sirolimus for the treatment of progressive kaposiform hemangioendothelioma: A multicenter retrospective study. Int J Cancer.

[21] Ji Y, Chen S, Zhou J, Yang K, Zhang X, Xiang B, Qiu T, Gong X, Zhang Z, Lan Y (2022). Sirolimus plus prednisolone vs sirolimus monotherapy for kaposiform hemangioendothelioma: a randomized clinical trial. Blood.

[22] Wang Z, Yang H, Xu X, Hu H, Bai Y, Hai J, Cheng L, Zhu R (2023). Ion elemental-optimized layered double hydroxide nanoparticles promote chondrogenic differentiation and intervertebral disc regeneration of mesenchymal stem cells through focal adhesion signaling pathway. Bioact Mater.

[23] Parsons JT, Slack-Davis J, Tilghman R, Roberts WG (2008). Focal adhesion kinase: targeting adhesion signaling pathways for therapeutic intervention. Clin Cancer Res.

[24] Belloni D, Heltai S, Ponzoni M, Villa A, Vergani B, Pecciarini L, Marcatti M, Girlanda S, Tonon G, Ciceri F (2018). Modeling multiple myeloma-bone marrow interactions and response to drugs in a 3D surrogate microenvironment. Haematologica.

[25] Ganguli A, Mostafa A, Saavedra C, Kim Y, Le P, Faramarzi V, Feathers RW, Berger J, Ramos-Cruz KP, Adeniba O, Diaz GJP, Drnevich J, Wright CL, Hernandez AG, Lin W, Smith AM, Kosari F, Vasmatzis G, Anastasiadis PZ, Bashir R. Three-dimensional microscale hanging drop arrays with geometric control for drug screening and live tissue imaging. Sci Adv. 2021;7(17):eabc1323. 10.1126/sciadv.abc1323 PMC8064630 33893093

[26] Nath S, Devi GR (2016). Three-dimensional culture systems in cancer research: Focus on tumor spheroid model. Pharmacol Ther.

[27] Verjans ET, Doijen J, Luyten W, Landuyt B, Schoofs L (2018). Three-dimensional cell culture models for anticancer drug screening: Worth the effort?. J Cell Physiol.

[28] Gevaert E, Dolle L, Billiet T, Dubruel P, van Grunsven L, van Apeldoorn A, Cornelissen R (2014). High throughput micro-well generation of hepatocyte micro-aggregates for tissue engineering. PLoS ONE.

[29] Li Y, Wu Q, Wang Y, Weng C, He Y, Gao M, Yang G, Li L, Chen F, Shi Y (2018). Novel spheroid reservoir bioartificial liver improves survival of nonhuman primates in a toxin-induced model of acute liver failure. Theranostics.

[30] Brill R, Uller W, Huf V, Muller-Wille R, Schmid I, Pohl A, Haberle B, Perkowski S, Funke K, Till AM (2021). Additive value of transarterial embolization to systemic sirolimus treatment in kaposiform hemangioendothelioma. Int J Cancer.

[31] Gruman A, Liang MG, Mulliken JB, Fishman SJ, Burrows PE, Kozakewich HP, Blei F, Frieden IJ (2005). Kaposiform hemangioendothelioma without Kasabach-Merritt phenomenon. J Am Acad Dermatol.

[32] Yeung J, Li W, Holinstat M (2018). Platelet Signaling and Disease: Targeted Therapy for Thrombosis and Other Related Diseases. Pharmacol Rev.

[33] Lyons LL, North PE, Mac-Moune Lai F, Stoler MH, Folpe AL, Weiss SW (2004). Kaposiform hemangioendothelioma: a study of 33 cases emphasizing its pathologic, immunophenotypic, and biologic uniqueness from juvenile hemangioma. Am J Surg Pathol.

[34] Glaser K, Dickie P, Dickie BH (2018). Proliferative Cells From Kaposiform Lymphangiomatosis Lesions Resemble Mesenchyme Stem Cell-like Pericytes Defective in Vessel Formation. J Pediatr Hematol Oncol.

[35] Ke ZY, Yang SJ (2017). Role of master transcriptional factor Prox-1 in lymphatic endothelial differentiation of Kaposiform hemangioendothelioma. Zhonghua Bing Li Xue Za Zhi.

[36] Folpe AL, Veikkola T, Valtola R, Weiss SW (2000). Vascular endothelial growth factor receptor-3 (VEGFR-3): a marker of vascular tumors with presumed lymphatic differentiation, including Kaposi's sarcoma, kaposiform and Dabska-type hemangioendotheliomas, and a subset of angiosarcomas. Mod Pathol.

[37] Yamashita M, Niisato M, Kawasaki Y, Karaman S, Robciuc MR, Shibata Y, Ishida Y, Nishio R, Masuda T, Sugai T, Ono M, Tuder RM, Alitalo K, Yamauchi K. VEGF-C/VEGFR-3 signalling in macrophages ameliorates acute lung injury. Eur Respir J. 2022;59(4):2100880. 10.1183/13993003.00880-2021 34446463

[38] Wang Y, Xing QF, Liu XQ, Guo ZJ, Li CY, Sun G (2016). MiR-122 targets VEGFC in bladder cancer to inhibit tumor growth and angiogenesis. Am J Transl Res.

[39] Kong D, Zhou H, Neelakantan D, Hughes CJ, Hsu JY, Srinivasan RR, Lewis MT, Ford HL (2021). VEGF-C mediates tumor growth and metastasis through promoting EMT-epithelial breast cancer cell crosstalk. Oncogene.

[40] Drolet BA, Trenor CC, Brandao LR, Chiu YE, Chun RH, Dasgupta R, Garzon MC, Hammill AM, Johnson CM, Tlougan B (2013). Consensus-derived practice standards plan for complicated Kaposiform hemangioendothelioma. J Pediatr.

[41] Wang Z, Li K, Yao W, Dong K, Xiao X, Zheng S (2015). Steroid-resistant kaposiform hemangioendothelioma: a retrospective study of 37 patients treated with vincristine and long-term follow-up. Pediatr Blood Cancer.

[42] Ji Y, Chen S, Li L, Yang K, Xia C, Li L, Yang G, Kong F, Lu G, Liu X (2018). Kaposiform hemangioendothelioma without cutaneous involvement. J Cancer Res Clin Oncol.

[43] Wang H, Guo X, Duan Y, Zheng B, Gao Y (2018). Sirolimus as initial therapy for kaposiform hemangioendothelioma and tufted angioma. Pediatr Dermatol.

[44] Wang Z, Yao W, Sun H, Dong K, Ma Y, Chen L, Zheng S, Li K (2019). Sirolimus therapy for kaposiform hemangioendothelioma with long-term follow-up. J Dermatol.

