# Supplementary material for: Construction and applications of the EOMA spheroid model of Kaposiform hemangioendothelioma
Source: J Biol Eng. 2024 Mar 14;18:21. doi: 10.1186/s13036-024-00417-4 (PMC10941415; doi:10.1186/s13036-024-00417-4)
Supplement: Supplementary file 1 — Supplementary Material 1. [file 13036_2024_417_MOESM1_ESM.docx]

**Construction and applications of the EOMA** **spheroid model of Kaposiform hemangioendothelioma**

Yanan Li^1, 2†^, Xinglong Zhu^3†^, Li Li^3^, Chunjuan Bao^3^, Qin Liu^3^, Ning zhang^1^, Ziyan He^1, 2†^, Yi Ji^1, 2*^, Ji Bao^3*^

^1^Division of Oncology, Department of Pediatric Surgery, West China Hospital of Sichuan University, Chengdu, 610041, China

^2^Med-X Center for Informatics, Sichuan University, Chengdu, 610041, China

^3^Department of Pathology, Institute of Clinical Pathology, Key Laboratory of Transplant Engineering and Immunology, NHC, West China Hospital, Sichuan University, Chengdu 610041, Sichuan Province, China

^†^These authors contributed equally to this work.

^*^Corresponding author:

Yi Ji, MD, PhD, E-mail: [jijiyuanyuan@163.com](mailto:jijiyuanyuan@163.com)

Division of Oncology, Department of Pediatric Surgery; West China Hospital of Sichuan University. 37# Guo-Xue-Xiang, Chengdu, 610041, China. Tel: +86 18980606865; Fax: +86 28 85423453.

Ji Bao, PhD, E-mail: baoji@scu.edu.cn

Department of Pathology, Institute of Clinical Pathology, Key Laboratory of Transplant Engineering and Immunology, West China Hospital, Sichuan University, 37# Guoxue Road, Chengdu 610041, Sichuan Province, China. Tel.: +86-28-85164030.

**
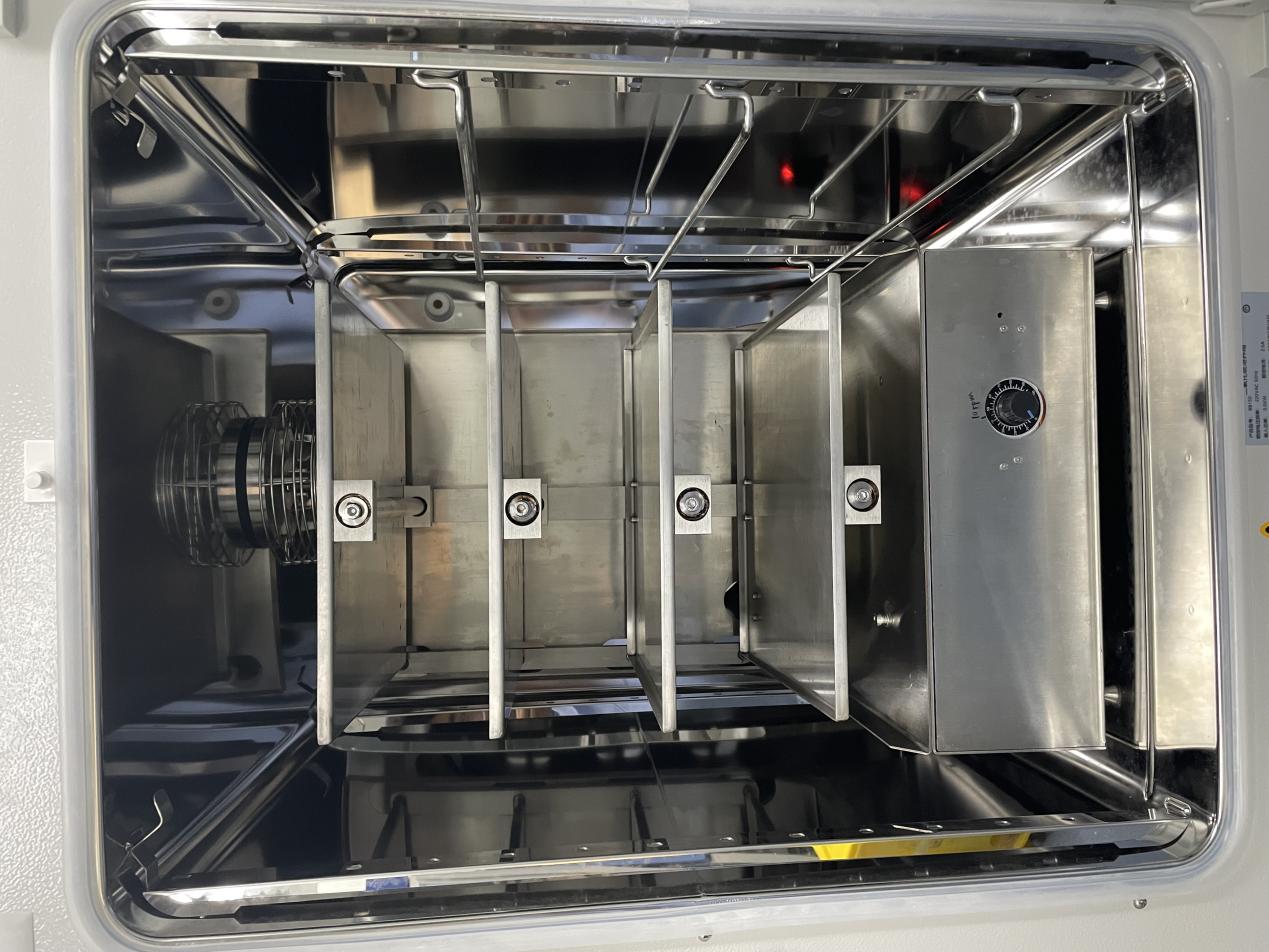
**

**Figure S1. The gross picture of RCCS.**


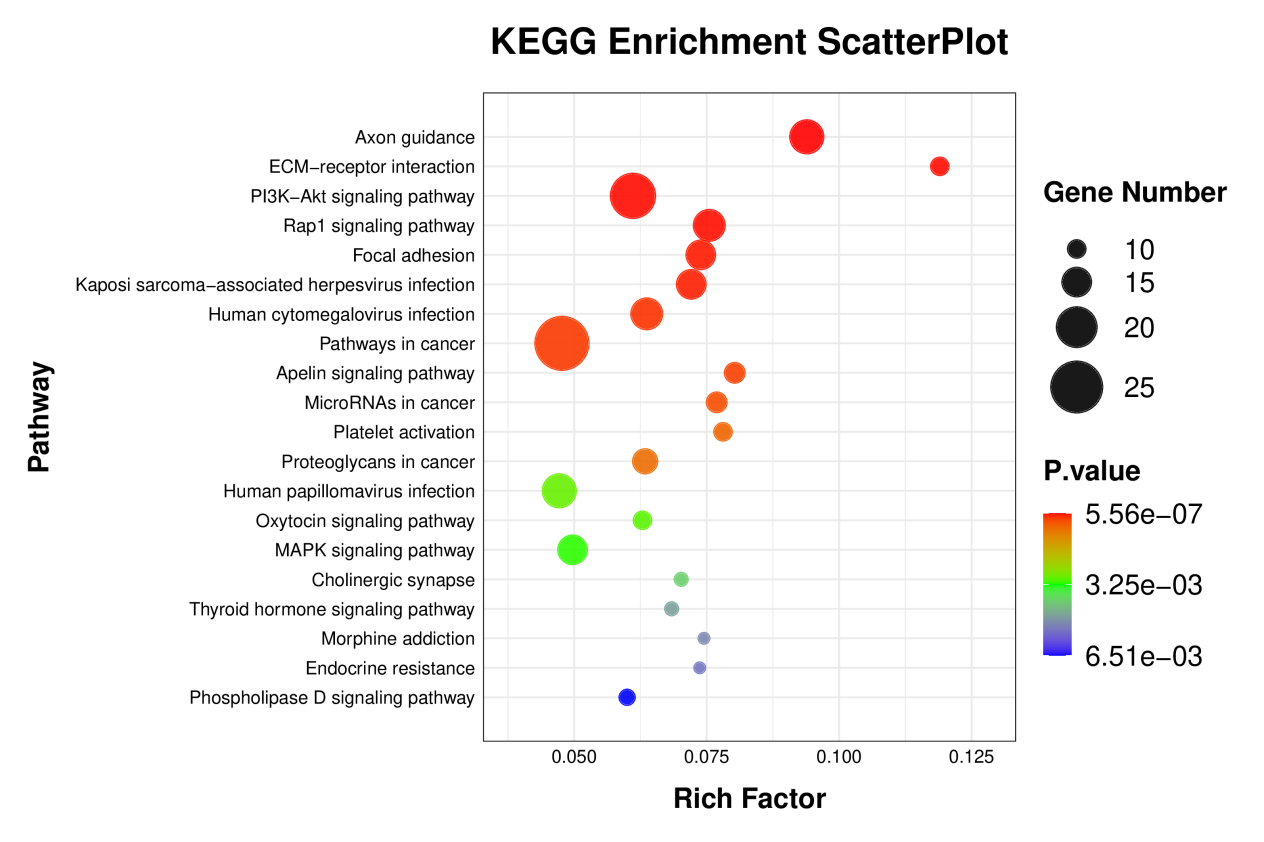


**Figure S2. GO enrichment analysis of 2D EOMA cells and 3D EOMA spheroids**

**Table S1. Primers used for qRT–PCR**

| Target | Forward | Reverse |
| --- | --- | --- |
| ITGB4 | CGGGATGAGGATGACGACTG | CGGGAGGGCAGTCTTTCTTT |
| FLT1 | CTGGATCCCAGCAGCAACTT | CACCAATGTGCTAACCGTCTTA |
| VEGFC | CTTGTCTCTGGCGTGTTCCC | GCCTTCAAAAGCCTTGACCT |
| TNXB | GGAGGAGCTGGTAAAAGGGC | GTCAGTCTGGCCTGTGCC |
| LAMA3 | AAGGCTACAGCAAGTCAGTCC | TGAGGTTGGCATCTAGCAGG |
| VWF | TGAGGTTGGCATCTAGCAGG | GAAACACTGCCACTCGGTTG |
| VEGFD | GAAACACTGCCACTCGGTTG | CTTCCAGTCCTCAGAGTGCG |
